# L2 speakers decompose morphologically complex verbs: fMRI evidence from priming of transparent derived verbs

**DOI:** 10.3389/fnhum.2014.00802

**Published:** 2014-10-10

**Authors:** Sophie De Grauwe, Kristin Lemhöfer, Roel M. Willems, Herbert Schriefers

**Affiliations:** ^1^Radboud University, Donders Institute for Brain, Cognition and BehaviourNijmegen, Netherlands; ^2^Max Planck Institute for PsycholinguisticsNijmegen, Netherlands

**Keywords:** language, fMRI, bilingual, morphological processing, priming, derivations

## Abstract

In this functional magnetic resonance imaging (fMRI) long-lag priming study, we investigated the processing of Dutch semantically transparent, derived prefix verbs. In such words, the meaning of the word as a whole can be deduced from the meanings of its parts, e.g., *wegleggen* “put aside.” Many behavioral and some fMRI studies suggest that native (L1) speakers decompose transparent derived words. The brain region usually implicated in morphological decomposition is the left inferior frontal gyrus (LIFG). In non-native (L2) speakers, the processing of transparent derived words has hardly been investigated, especially in fMRI studies, and results are contradictory: some studies find more reliance on holistic (i.e., non-decompositional) processing by L2 speakers; some find no difference between L1 and L2 speakers. In this study, we wanted to find out whether Dutch transparent derived prefix verbs are decomposed or processed holistically by German L2 speakers of Dutch. Half of the derived verbs (e.g., *omvallen* “fall down”) were preceded by their stem (e.g., *vallen* “fall”) with a lag of 4–6 words (“primed”); the other half (e.g., *inslapen* “fall asleep”) were not (“unprimed”). L1 and L2 speakers of Dutch made lexical decisions on these visually presented verbs. Both region of interest analyses and whole-brain analyses showed that there was a significant repetition suppression effect for primed compared to unprimed derived verbs in the LIFG. This was true both for the analyses over L2 speakers only and for the analyses over the two language groups together. The latter did not reveal any interaction with language group (L1 vs. L2) in the LIFG. Thus, L2 speakers show a clear priming effect in the LIFG, an area that has been associated with morphological decomposition. Our findings are consistent with the idea that L2 speakers engage in decomposition of transparent derived verbs rather than processing them holistically.

## INTRODUCTION

During the past few decades, the processing of morphologically complex words has led to considerable debate. Many studies have been devoted to the question whether these words are decomposed into their constituent parts or processed holistically. Semantically transparent derivations (e.g., *reread*, derived from *read*) provide an interesting case in this debate. On the one hand, they differ from semantically opaque derivations (e.g., *understand*, derived from *stand*) in terms of meaning compositionality: their meaning as a whole is related to the meaning of their constituent parts, in contrast with opaque derivations, whose meaning cannot be inferred from the meaning of their parts. Thus, lexical access to transparent derivations might be accomplished by decomposition of these words into their constituent parts. On the other hand, transparent derivations differ from inflections (e.g., *reads*, the present tense third person singular form of *read*), in that they, like opaque derivations, are the result of historical word formation processes, whereas inflections are the result of syntactic operations. Thus, transparent derivations constitute new words, in contrast with inflections, which constitute different forms of the same word. As a result, transparent derivations might be associated with full lexical entries in the so-called “mental lexicon,” potentially leading to holistic processing of these complex words (see, for example, Marslen-Wilson, 2007, for a discussion of this issue).

As we will see below, the majority of the available evidence suggests that native (L1) speakers decompose transparent derivations. This makes transparent derivations a particularly interesting test case for the processing of transparent derivations in non-native (L2) speakers, as one could hypothesize that L2 speakers may not (yet) have grasped the compositionality of these words, and thus tend to process them holistically (see, for example, Clahsen et al., 2010). Most studies on the processing of transparent derivations have tested (especially L1) speakers in behavioral tasks. In this study, we use functional magnetic resonance imaging (fMRI) to investigate the neural correlates of the processing of semantically transparent derivations in L2 speakers.

Many behavioral studies on L1 processing of transparent derivations have used the morphological priming/lexical decision method. In this approach, a target word is preceded by a morphologically related word or an unrelated word. For example, a morphologically complex word such as *reread* is preceded by its stem (*read*), or vice versa. Participants have to decide as quickly as possible whether the target is a real word or not (lexical decision task). In visual priming (targets and primes presented visually), primes and targets may be separated by several intervening stimuli (long-lag priming) or follow each other without intervening stimuli (short-lag priming) ^1^. The underlying idea is that if *reread* and *read* are separate entries in the mental lexicon, *read* should not facilitate the recognition of *reread* any more than a control prime like *think* does. In contrast, if the recognition of the target word *reread* involves its decomposition into *re-* and *read*, the previous encounter with one of these parts (*read*) should speed up recognition. The results of these studies mostly show significant facilitatory priming for transparent derivations in L1 speakers, both in long-lag priming (Napps, 1989; Raveh and Rueckl, 2000; Rueckl and Aicher, 2008) and in short-lag priming (Feldman and Soltano, 1999; Rastle et al., 2000; Feldman et al., 2002, 2004; Smolka et al., 2009, 2014). These results have been interpreted as evidence that transparent derivations are decomposed during lexical access.

However, the interpretation of priming effects with transparent derivations is complicated by the fact that transparent derivations are not only morphologically, but also semantically and formally related to their stems. Thus, the observed priming effects could be due to the semantic and/or form overlap between transparent derivations and their stems, rather than to their morphological relationship. However, long-lag priming typically elicits facilitatory effects of morphological relatedness, but not of semantic or form relatedness (Napps and Fowler, 1987; Napps, 1989; Feldman, 2000; Rueckl and Aicher, 2008, Experiment 1). For example, in a series of long-lag priming experiments, morphologically related word pairs such as *manager–manage* led to significant facilitatory priming, whereas no priming was found for form-related (e.g., *ribbon–rib*) or semantically related (e.g., *ache–pain*) word pairs (Napps and Fowler, 1987; Napps, 1989). Therefore, long-lag priming seems particularly useful for the study of transparent derivations: any facilitatory priming effects for transparent derivations in long-lag priming will likely be due to the morphological relationship of the prime-target pair rather than their semantic or form relationship.

In fMRI studies on the processing of transparent derivations, the left inferior frontal gyrus (LIFG) has often been associated with morphological decomposition of these words. For example, in two lexical decision fMRI studies (Meinzer et al., 2009; Pliatsikas et al., 2014b), increased LIFG activation was found for morphologically complex compared to morphologically less complex semantically transparent words. The two conditions were matched on a number of lexical and semantic characteristics, such as length, frequency, concreteness, etc., and only differed in degree of derivational complexity. In both studies, the authors therefore concluded that transparent derivations are decomposed, and that this decomposition process is supported by the LIFG (see also Vannest et al., 2005, 2011, for similar results for “decomposable” vs. “non-decomposable” derived words and for derived vs. simple words, respectively; but see Davis et al., 2004; Bozic et al., 2013, who found no selective activation of the LIFG for derived vs. simple words).

The fMRI studies mentioned so far did not use morphological priming. In contrast, Bozic et al. (2007) used a long-lag priming paradigm in an fMRI study contrasting morphologically, semantically and form-related word pairs. In fMRI studies, priming often leads to “repetition suppression”: a decrease in the BOLD response to primed compared to unprimed targets. This decrease is supposed to reflect faster or “more efficient” processing of the primed target in a certain brain region, due to the application of the same processes in that brain region as during exposure to the prime (Schacter and Badgaiyan, 2001; Henson, 2003). In the unprimed condition, the same process is supposed to operate on the stimulus, but in this case, processing is not facilitated by the earlier presentation of a prime – there is no prime “greasing the tracks,” so to say (Henson, 2003). Thus, if a brain area such as the LIFG displays a decreased hemodynamic response to a morphologically complex word that is primed by its stem, this is an indication that, in this brain region, processing of the complex word involves processing of its stem – suggesting that the complex word is morphologically decomposed. This is precisely what Bozic et al. (2007) found: the LIFG showed lower activation for target words primed by morphologically related primes than for unprimed target words. This was not the case for semantically or form-related prime-target pairs, indicating that the long-lag priming effect was not due to the overlap between form and meaning. The LIFG therefore seemed to be specifically involved in morphological processing.

Several other brain areas have been implicated in the processing of derivations, such as the right inferior frontal gyrus (Bick et al., 2010; Bozic et al., 2013), middle temporal cortex (Meinzer et al., 2009; Bozic et al., 2013), superior temporal cortex (Meinzer et al., 2009; Vannest et al., 2011; Bozic et al., 2013), inferior temporal and occipital-temporal cortex (Bick et al., 2010), and occipital cortex (Meinzer et al., 2009; Bick et al., 2010). However, only a minority of fMRI studies on derivation processing report evidence of their involvement, in contrast with the more consistent evidence that exists for the involvement of the LIFG. This is why, as we will see later, we conducted region of interest (ROI) analyses of the LIFG only, whereas the potential involvement of other brain areas was assessed through whole-brain analyses.

Only a few behavioral studies have been conducted on the processing of transparent derivations in L2 speakers. To our knowledge, all of them used other paradigms than unmasked priming. These studies have produced conflicting results. In a masked priming experiment, Clahsen and Neubauer (2010) found no priming effect for morphologically related prime-target pairs (German derived nouns and their stems) in Polish L2 speakers of German, as opposed to L1 speakers of German. In another masked priming study, Silva and Clahsen (2008) found that priming was reduced for morphologically related prime-target pairs (English derived nouns and their stems) compared to word pairs with identical prime and target in Chinese and German L2 speakers of English. In contrast, L1 speakers of English showed similar effects for morphological and identical priming. The results of these experiments were interpreted as suggesting that L2 speakers relied more on holistic processing than L1 speakers.

Other studies, however, report no differences between L1 and L2 speakers in terms of the processing of transparent derivations. Diependaele et al. (2011) also used masked priming, and found similar facilitatory priming effects for transparent derivations in L1 speakers of English and in L2 speakers of English (with either Spanish or Dutch as their L1). These results suggest that both native speakers and bilinguals decomposed the complex words (see also Kirkici and Clahsen, 2013, for similar results for derivations in their masked priming experiment with L2 speakers of Turkish). In an unprimed visual lexical decision study, Portin and Laine (2001) found that both L1 speakers of Swedish and early Finnish–Swedish bilinguals showed shorter lexical decision latencies to transparent derived nouns than to morphologically simple nouns of the same length and frequency. One of the possible interpretations discussed by the authors refers to parallel dual-route models (more specifically the morphological race model proposed in Frauenfelder and Schreuder, 1992). According to this interpretation, transparent derivations might be processed faster because of a race between two parallel lexical access routes (a decompositional route and a whole-word route). In contrast, simple nouns can only be processed through the whole-word route, and thus would not benefit from the race between two competing routes.

The conflicting evidence reported in these behavioral studies may be due to differences in paradigms: masked priming (Silva and Clahsen, 2008; Clahsen and Neubauer, 2010; Diependaele et al., 2011) vs. unprimed lexical decision (Portin and Laine, 2001); materials: homogeneous (Portin and Laine, 2001; Silva and Clahsen, 2008; Clahsen and Neubauer, 2010) vs. inhomogeneous (Diependaele et al., 2011) in terms of suffix and/or word class of derived words, matched vs. unmatched in terms of length and/or frequency of derived and unrelated primes (Silva and Clahsen, 2008: prime length not matched, no information on whole-word prime frequency; Clahsen and Neubauer, 2010: no information on prime frequency); participants: early (Portin and Laine, 2001) vs. late (Silva and Clahsen, 2008; Clahsen and Neubauer, 2010; Diependaele et al., 2011) bilinguals; and/or differences in L1–L2 combinations (Clahsen and Neubauer, 2010: Polish-German; Silva and Clahsen, 2008: Chinese/German-English; Diependaele et al., 2011: Spanish/Dutch-English; Portin and Laine, 2001: Finnish–Swedish).

In the fMRI literature, to our knowledge, only three studies have addressed morphological processing in L2 speakers: two on inflectionally complex words (Lehtonen et al., 2009; Pliatsikas et al., 2014a) and one on derivations (Bick et al., 2010). In all three studies, the LIFG was associated with morphological processing. Lehtonen et al. (2009) used an unprimed visual lexical decision task with early Finnish–Swedish bilinguals. Each participant saw two lists of simple and inflected nouns: a Swedish list and a Finnish list. The results showed increased activation of the LIFG for Finnish inflected nouns compared to Swedish inflected nouns and to Finnish simple nouns, suggesting decomposition in Finnish and holistic processing in Swedish. This was linked to the structural difference between Finnish (morphologically rich) and Swedish (morphologically poor). Pliatsikas et al. (2014a) used a masked priming task involving inflected verbs with late Greek L2 learners of English. They found activation in a network including the LIFG for morphologically related regular verb pairs compared to morphologically related irregular verb pairs (which are more likely to be represented holistically) and to unrelated regular verb pairs. This pattern of results was found for the combined group of L1 and L2 speakers of English, with no indication of any between-group differences. Therefore, the L2 speakers were interpreted to use the same decompositional strategy as the L1 speakers. Masked priming was also used by Bick et al. (2010) in their study of derivational processing in early Hebrew–English bilinguals. A bilateral network including the LIFG was found to show lower activation for morphologically related prime-target pairs compared to semantically related and orthographically related prime-target pairs. This repetition suppression effect was found for both Hebrew and English transparent derivations, suggesting decomposition in both languages. Although all three studies found evidence for the involvement of the LIFG in L2 morphological processing, none of them contrasted L1 and L2 processing of transparent derivations. The neural correlates of derivational processing in late bilinguals remain to be investigated.

With this study, we want to find out whether transparent derivations are decomposed or processed holistically in late bilinguals. Decomposition may be challenging for L2 speakers because it requires an understanding of the morphological structure of words – an understanding which may develop only after extended experience with the language. However, holistic processing also comes at a cost, as it requires extended memory resources for the storage of whole-word forms. The behavioral evidence on this issue is mixed. By using fMRI, this study may shed new light on derivational processing in late bilinguals.

The stimuli used in this experiment consisted of two types of prefix verbs, i.e., particle verbs (verbs with separable particles, e.g., *meenemen* “take along”) and prefixed verbs (verbs with non-separable particles, e.g., *omvatten* “enclose”). Particle verbs differ from prefixed verbs in that their particles are separated from their stem when used in finite form in main clauses (e.g., *Zij* ***neemt*** *het boek* ***mee*** “She takes the book along”). One could hypothesize that, because of their separability, particle verbs are more likely to be morphologically decomposed than prefixed verbs. However, several studies comparing the two types of prefix verbs have found no processing differences between prefixed and particle verbs in terms of decomposition (Schriefers et al., 1991; Lüttmann et al., 2011). For this reason, both types of stimuli were used in this study. Care was taken that the proportion of each type was balanced over conditions.

In this fMRI study, we contrasted native speakers of Dutch with late learners of Dutch who had German as their L1. Using long-lag priming, the processing of semantically transparent derived verbs was investigated in both groups. We wanted to determine whether L1 and L2 speakers show a repetition suppression effect for morphologically primed vs. unprimed derived verbs in the LIFG in particular. We expected this to be the case for L1 speakers, thus replicating Bozic et al.’s (2007) results. For L2 speakers, no clear prediction can be formulated on the basis of the mixed existing literature. If L2 speakers decompose transparent derived verbs, we should also find an LIFG repetition suppression effect for derived verbs primed by their stems. If they process these verbs holistically, we should not find such an effect.

Since we had a clear prediction for the involvement of the LIFG in derivation processing (at least in L1 speakers), we used ROI analyses to investigate effects in this area. Regarding the involvement of other brain areas, predictions were less clear, because of the inconsistency in the existing literature on derivation processing. However, because there is at least some evidence that brain areas such as temporal cortex may be involved, we also conducted whole-brain analyses. In this way, we made sure not to miss effects in brain areas less attested in the literature.

The present study was the second part of a two-part fMRI session^2^. Each part of this session constituted an experiment on its own. The results of the first part are reported in De Grauwe et al. (2014). The second part provided the data reported in the current study. In the description of the methods used, the reader is referred to De Grauwe et al.’s (2014) study where appropriate.

As mentioned above, a long-lag priming methodology was used. Complex transparent verbs (targets) were preceded by their stems (primes), with four to six intervening stimuli (primed condition). This condition was contrasted with a condition with complex verb targets that were not preceded by their stem (unprimed condition). To keep the set of stimuli similar across the two priming conditions, the verb targets in the unprimed condition were followed by their stem, with the same number of intervening stimuli. The potential priming effect in the primed condition was enhanced by making use of part 1 of the two-part fMRI session: in addition to its presentation as a prime for the primed complex verb target in part 2, the stem had already been presented twice in part 1, once as a simple verb and once as the stem of a semantically opaque complex verb. Thus, primed complex targets were primed three times: twice in part 1 and once in part 2. In contrast, the stems of unprimed complex targets had not been presented before (neither in part 1 nor 2). An overview of the design can be found in **Table 1**.

**Table 1:** Design. Triple priming vs. no priming.

		Part 1	Part 2
**Words**	**Primed**	nemen – ondernemen *(nehmen/take – unternehmen/undertake)*	nemen – **meenemen** *(nehmen/take – mitnehmen/ take along)*
	**Unprimed**	–	**inslapen** – slapen *(einschlafen/fall asleep – schlafen/sleep)*
**Pseudo-Words**	**Primed**	ralmen – verralmen	ralmen – **verralmen**
	**Unprimed**	–	**bemelgen** – melgen

German and English translations in parentheses. Targets are printed in bold.

## MATERIALS AND METHODS

### PARTICIPANTS

Initially, 21 L1 speakers^3^ of Dutch and 29 German L2 speakers of Dutch participated in the study. After exclusion (for details, see Results below), 18 L1 speakers (14 female, four male) and 21 L2 speakers (13 female, eight male) remained. The mean age of the remaining participants was 22.11 (SD: 2.42, range 18–26) for L1 speakers and 24.62 (SD: 2.13, range 22–29) for L2 participants.

The L2 participants, most of them students at the Radboud University Nijmegen, had German as their dominant language, had lived and/or studied in the Netherlands for at least 1.5 years, and used Dutch regularly for their studies, work and/or private life. Prior to the fMRI experiment, they were asked to complete the online version of the Dutch LexTALE test (Lemhöfer and Broersma, 2012), a non-speeded visual lexical decision test. Only participants with a minimum score of 67.50% were invited for the fMRI experiment. The average score of the selected participants on the LexTALE test was 78.04% (SD 7.63%). After participating in the fMRI experiment, L2 participants completed a self-assessment rating on their proficiency in Dutch (see Supplementary Material, Table S1, for results). Their mean age of acquisition of Dutch was 20.10 (SD 2.45), and they had an average of 4.52 (SD 3.03) years of experience with Dutch.

The L1 participants, most of them students at the Radboud University Nijmegen, had Dutch as their first and dominant language. They had lived in the Netherlands from birth.

All participants were right-handed and reported having no reading disorders. They gave their written consent in accordance with national legislation and the Helsinki Declaration of 1975, revised in 2004. The study received ethical approval from the local reviewing committee (Commissie Mensgebonden Onderzoek, regio Arnhem Nijmegen; approval number 2001/095 and amendment “Imaging Human Cognition” 2006, 2008).

### MATERIALS

Seventy Dutch morphologically complex verbs were selected as targets (see **Table 1** for examples). They were all semantically transparent, derived Dutch prefix verbs. Because of the high similarity between Dutch and German, it was not possible to select enough non-cognate verbs of this type. Therefore, we restricted ourselves to cognate verbs. These were mostly non-identical in form (e.g., *inslapen* – German: *einschlafen*/English: *fall asleep*), except for two verbs (*bedienen* – German: *bedienen*/English: *serve*; *bemerken* – German: *bemerken*/English: *notice*). Half of the targets occurred in the primed condition, the other half in the unprimed condition. The primed condition contained 28 particle (i.e., separable) verbs and seven prefixed (i.e., non-separable) verbs, whereas the unprimed condition contained 27 particle verbs and eight prefixed verbs.

Complex targets were selected on the basis of two prior rating studies. First, the degree of transparency of the complex verbs was determined on the basis of the transparency/opacity rating reported by De Grauwe et al. (2014). Primed and unprimed transparent complex verbs were matched on degree of transparency, as determined by a *t*-test (*p* > 0.47). Second, De Grauwe et al. (2014) had selected stems such that they were either clearly motor-related or not. Thus, the stems of the primed complex targets in the current study were either clearly motor-related or not. To match these stems with the stems of the unprimed complex targets (which did not occur in De Grauwe et al., 2014), the same number of motor- and non-motor-related stems was included in both priming conditions (19 motor-related and 16 non-motor-related stems in each condition). In addition, the degree of motor-relatedness was rated (see De Grauwe et al., 2014) and matched for stems in the primed and unprimed conditions (*p* > 0.66). Primed and unprimed complex verbs were also matched in terms of whole-word length and stem length (number of letters; *p*s > 0.53), and whole-word frequency and stem frequency (log-transformed lemma frequency, based on the Celex database, Baayen et al., 1995; *p*s > 0.39). (See Supplementary Material, Table S2, for further details on stimulus characteristics).

Thus, participants saw 140 words: 35 primed complex targets, 35 unprimed complex targets, 35 stems used as primes for the primed complex targets, and 35 stems used as fillers (following the complex targets in the unprimed condition). Twenty-eight pseudo-words were added, all of them verb-like (ending in the Dutch infinitive suffix “en”) and obeying the phonotactic rules of Dutch. They were created by changing one or more letters of real Dutch words. Half of them were “complex,” consisting of an existing Dutch prefix and a non-existing stem. The other half were “simple,” being the non-existing stems of the complex pseudo-words. Half of the complex pseudo-words were “primed,” that is they were preceded by their stem in the present study (i.e., in part 2 of the fMRI session) and had also been presented in part 1 of the fMRI session (see **Table 1**). The other half of the complex pseudo-words were “unprimed.”

### STIMULUS PRESENTATION

Participants saw the stimuli through a mirror attached to the head coil while lying on their back in the scanner. Their task was to respond to pseudo-words only (go/no-go task), by pushing a button on a response box with their right index finger. Each trial started with a blank screen presented for a variable jitter time (0–2000 ms), followed by a fixation cross (400 ms). Then the stimulus appeared and remained on the screen for 2000 ms or until a response was recorded. Finally, a blank screen was presented until the fixed trial length of 8440 ms was reached. Word and pseudo-word trials were interspersed with 28 null trials. These consisted of a blank screen shown for 8440 ms. The stimuli were presented in 20-point, light-gray, lower-case letters in Arial font against a black background using Presentation software (developed by Neurobehavioral Systems, ).

Four different lists were generated. Each list was randomized with the restriction that words of the same word condition and pseudo-words were not presented on more than three consecutive trials. Primed complex verbs were always preceded by their stem, while unprimed complex verbs were always followed by their stem, with four to six intervening stimuli between a complex verb and its stem in both cases. Participants saw all 196 trials in one block, which lasted ∼30 min.

Before the fMRI session, participants were familiarized with the task in a practice block of eight word and eight pseudo-word trials outside the scanner. Following the fMRI session, they completed two off-line ratings: a motor-relatedness rating of the words of part 1 (see De Grauwe et al., 2014) and a familiarity rating of the words of part 2. In the familiarity rating, participants were asked to indicate for each word if they knew it or not. Finally, L2 participants filled out a language background questionnaire to rate their proficiency in Dutch (see Supplementary Material, Table S1, for results).

### BEHAVIORAL DATA ANALYSIS

Mean error percentages to words and pseudo-words were calculated. Error percentages to complex words were analyzed with a 2 × 2 repeated-measures analysis of variance (ANOVA) with the factors of Language (between-participant factor; L1 vs. L2) and Priming (within-participant factor; Primed vs. Unprimed).

Participants were excluded from further analysis if they made more than 30% errors to pseudo-words or if less than 25 trials per critical condition remained in the fMRI analysis. Items were excluded from further analysis for a certain language group if their error percentage was more than three standard deviations above the mean of their language group. Only correctly answered trials were included in the fMRI analyses.

### fMRI DATA ACQUISITION AND ANALYSIS

Whole-brain images were acquired on a Siemens TRIO 3.0T MRI system (Siemens, Erlangen, Germany). For the EPI images, the following acquisition parameters were used: 31 axial slices, TR = 2110 ms, TE = 30 ms, flip angle = 90^∘^, voxel size = 3.5 mm × 3.5 mm × 3.5 mm. High-resolution anatomical images were acquired using an MPRAGE sequence (192 sagittal slices, TR = 2300 ms, TE = 3.03 ms, FOV = 256, voxel size = 1 mm × 1 mm × 1 mm).

Imaging data were analyzed using SPM8 (Statistical Parametric Mapping, ). After discarding the first five volumes, preprocessing was performed by motion correction through rigid body registration along three translations and three rotations, slice timing correction using the middle slice (slice 17) as reference, normalization to the T1 image in MNI space and spatial smoothing using an isotropic 8-mm FWHM Gaussian kernel. For one participant, the normalization procedure led to considerable distortion. Therefore, this participant’s images were normalized to a standard EPI template centered in MNI space.

For the first-level analysis, the preprocessed functional images of each participant were analyzed using the general linear model with regressors for each word condition (Primed, Unprimed, Stem Prime, and Stem Filler). A regressor for the null trials was added, as well as the six realignment parameters generated during motion correction (three translation and three rotation parameters). The regressors were convolved with a canonical hemodynamic response function.

#### ROI analyses

To find out whether primed complex verbs (compared to unprimed complex verbs) led to repetition suppression in the LIFG, three ROIs were defined in this area: Brodmann Area (BA) 44, 45, and 47. For this, the BAs section of the Talairach Daemon database was used in the WFU PickAtlas toolbox (Lancaster et al., 1997, 2000; Maldjian et al., 2003, 2004). Together, these three ROIs make up the most part of LIFG gray matter. Using these ROIs thus allows us to derive conclusions regarding activation in the LIFG ROIs separately (if an interaction with the ROI factor is found) or regarding activation in the LIFG as a whole (if effects found are not modulated by the ROI factor).

For each participant and each ROI, the contrast values for each complex verb condition compared to the null condition were calculated using MarsBar, and averaged across all voxels in the ROI (Brett et al., 2002). These were entered into a (3 × 2 × 2) repeated-measures ANOVA with the factors of ROI (BA44 vs. BA45 vs. BA47), Language (L1 vs. L2) and Priming (Primed vs. Unprimed). In addition, results for each language group were analyzed separately using repeated-measures ANOVAs with the factors of ROI (BA44 vs. BA45 vs. BA47) and Priming (Primed vs. Unprimed). Only effects and interactions involving Priming are reported. A significance level of α = 0.05 was used, and the Greenhouse and Geisser (1959) correction was applied to correct for violations of sphericity when there was more than one degree of freedom in the numerator. In those cases, original degrees of freedom and adjusted *p*-values are reported.

#### Whole-brain analyses

To determine whether other brain regions are also involved in the processing of morphologically complex words, we conducted a second-level random effects analysis over both language groups. For this, the contrast images of the complex word conditions vs. the null condition of each participant were entered into a full-factorial 2 × 2 analysis (Language: L1 vs. L2; Priming: Primed vs. Unprimed). The main effect of Priming and the interaction between Language and Priming were investigated with directional *t*-tests: Unprimed – Primed and reverse, and L1 (Unprimed – Primed) – L2 (Unprimed – Primed) and reverse, respectively. In addition, *t*-tests were used to investigate whether the effect of Priming was present for each of the two language groups separately.

A double threshold was used to protect against false positives: a voxel-level *p*-value of *p* < 0.005 (uncorrected) was combined with a minimum cluster size of 65 voxels. This led to a correction for multiple comparisons of *p* < 0.05, as determined by the randomization method proposed by Slotnick et al. (2003; see also De Grauwe et al., 2014, for more details).

## RESULTS

Eight (one L1, seven L2) out of the original 50 participants were excluded because their number of errors exceeded the criteria set. One additional L2 participant was excluded because of excessive motion, and two additional L1 participants were excluded because of compromised data quality. For each language group, three items were excluded because their percentage of errors exceeded the criterion set (see Supplementary Material, Table S3, for details).

### BEHAVIORAL RESULTS

On average, the L1 participants only made 1.5% errors to words (SD 1.6%) and 4.2% errors to pseudo-words (SD 4.8%). L2 participants made 5.2% errors to words (SD 4.1%) and 11.9% errors to pseudo-words (SD 8.7%), indicating that, as to be expected, the task was more demanding for them.

**Table 2** gives the mean error percentages for complex verbs for L1 and L2 speakers. The repeated-measures ANOVA on the error percentages on complex verbs revealed significant main effects of Language [*F*(1,17) = 9.52, *p* < 0.01] and Priming [*F*(1,17) = 8.16, *p* < 0.01], modulated by a significant Language by Priming interaction [*F*(1,17) = 6.20, *p* < 0.05]. Follow-up analyses for the two language groups separately showed that L2 speakers made fewer errors to primed than to unprimed complex verbs (*p* = 0.001), whereas no difference was found between the two conditions in L1 speakers (*p* > 0.79).

**Table 2:** Behavioral results. Mean error percentages to complex verbs.

	L1 speakers	L2 speakers
Primed	2.3 (3.7)	4.9 (4.7)
Unprimed	2.5 (2.8)	8.4 (6.5)

Standard deviation in parentheses.

### fMRI RESULTS

#### ROI analyses

The ANOVA over both groups revealed that the main effect of Priming was significant, indicating that primed complex verbs elicited less activation in the LIFG than unprimed complex verbs (**Table 3**). None of the interactions of Priming with the other two variables (Language and ROI) was significant.

**Table 3:** ROI analyses. Repeated-measures ANOVAs on contrast values for complex verbs.

	Both groups			L1 speakers			L2 speakers		
Effect	*df*	*F*	*p*	*df*	*F*	*p*	*df*	*F*	*p*
Priming	1,37	6.72	0.014	1,17	1.01	0.33	1,20	6.67	0.018
ROI × Priming	2,74	0.12	0.82	2,34	0.17	0.77	2,40	0.80	0.42
Language × Priming	1,37	1.96	0.17	–	–	–	–	–	–
Language × ROI × Priming	2,74	0.83	0.41	–	–	–	–	–	–

*–, not applicable.*

**FIGURE 1:**
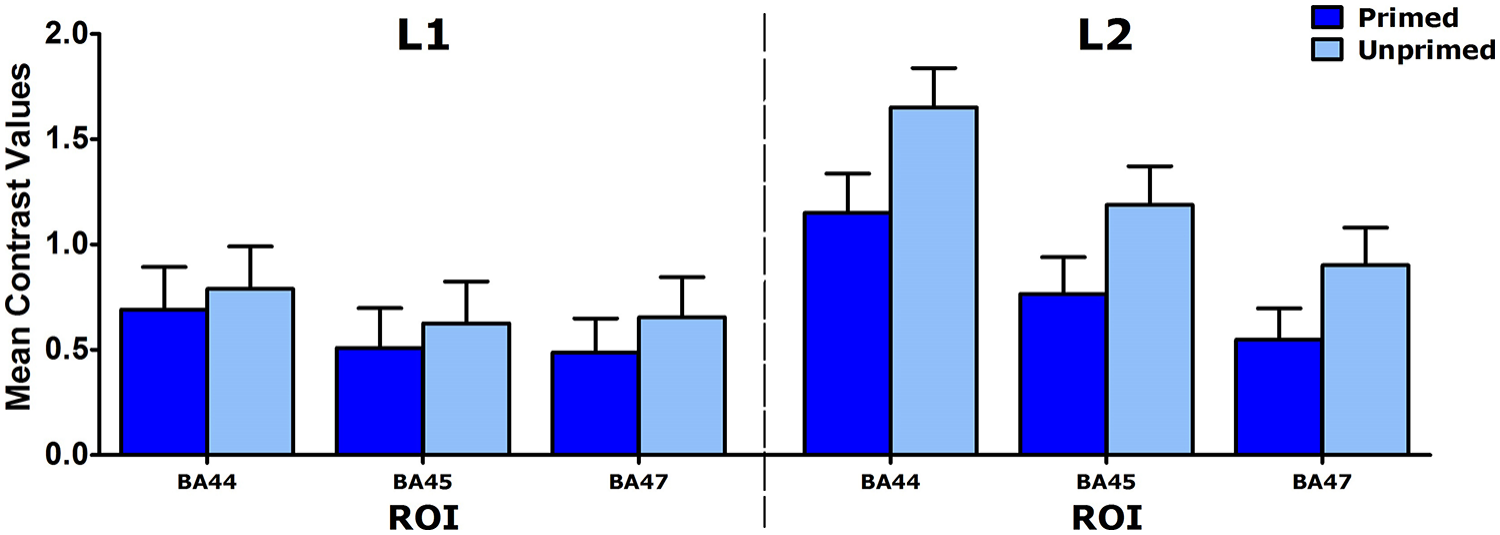
**Mean contrast values for three LIFG ROIs (BA44, BA45, and BA47) for L1 and L2 speakers.** Error bars: +1 SE.

Although the interactions involving Priming and Language were not significant, L1 and L2 speakers were also analyzed separately for exploratory purposes, to make sure that the Priming effect was indeed present in both groups (see **Figure 1**). The ANOVA for L2 speakers showed that the LIFG was activated less for primed than for unprimed complex verbs. For L1 speakers, however, no such difference was found: none of the effects or interactions was significant.

To determine whether the null hypothesis (i.e., no difference between primed and unprimed complex verbs) can be accepted for L1 speakers, we performed a Bayesian analysis of the L1 data. For this, we used Masson’s (2011) approach, which is based on a transformation of the sum-of-squares values obtained in a regular ANOVA. For the main effect of Priming with L1 speakers, the resulting Bayes factor was 2.53. This is equivalent to 71.6% support for the null hypothesis, as opposed to 28.4% support for the alternative hypothesis. According to Raftery (1995), this constitutes weak evidence in favor of the null hypothesis.

We also wanted to know whether the neural priming effect found for L2 speakers is due to the increased difficulty of unprimed compared to primed complex verbs. Therefore, a regression analysis was performed. The predictor in this analysis was the difference in error percentage between unprimed and primed complex verbs for L2 participants. For the dependent variable, an LIFG ROI was created by combining the BA44, BA45 and BA47 ROIs. For this ROI, contrast values were extracted for primed and unprimed conditions for each L2 participant using MarsBar (Brett et al., 2002). The difference between the contrast values for unprimed and primed complex verbs constituted the dependent variable. Results showed no evidence that the size of the priming effect in error percentages predicted the difference in contrast values between unprimed and primed conditions (*p* = 0.84).

So far, the results indicate that L2 participants show a clear Priming effect for complex verbs in the LIFG. The results for L1 participants are not as clear: descriptively, they also show a Priming effect, and the analysis over both groups shows no evidence of an interaction of the significant Priming effect with participant group. However, in the analysis over L1 speakers only, the Priming effect fails to reach significance. Still, the Bayesian analysis of the L1 results only provides weak evidence for the absence of a Priming effect. These results will be addressed in more detail in the Discussion.

#### Whole-brain analyses

To examine whether the Priming effect was present not only in the LIFG but also in other brain regions, a full-factorial second-level analysis over both groups was performed (see Supplementary Material, Table S4, for an overview of significant activations).

**FIGURE 2:**
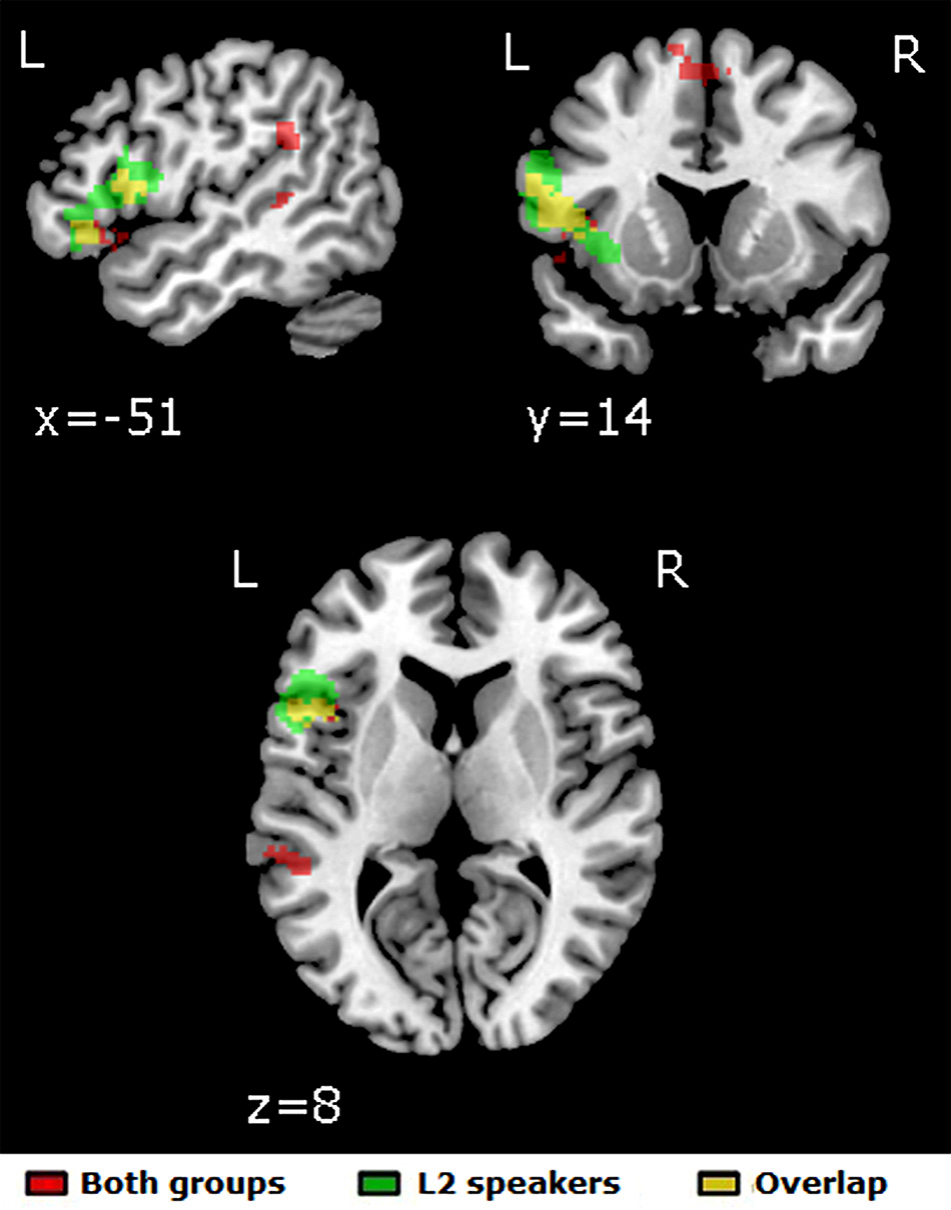
**Significant clusters of activation for the [Unprimed – Primed] contrast in the full-factorial whole-brain analysis.** Red: both groups; green: L2 speakers; yellow: overlap between activations for both groups and for L2 speakers. *p* < 0.005/k > 65, leading to a correction for multiple comparisons of *p* < 0.05. No significant activation was found for L1 speakers for this contrast.

The Unprimed vs. Primed contrast yielded five significant left-lateralized clusters of activation: from the pars orbitalis to the pars triangularis in the LIFG (overlapping with the BA47 ROI), in the pars opercularis of the LIFG (overlapping with the BA44 and some of the BA45 ROI) reaching into the insula, in the supramarginal gyrus, in the posterior superior temporal sulcus and in the bilateral medial superior frontal gyrus (see **Figure 2**).

The reverse contrast (Primed vs. Unprimed) also revealed five significant clusters: one cluster extended from the left insula to the left superior temporal gyrus, one was found in the right superior temporal gyrus, one in the right hippocampus reaching into the parahippocampal gyrus, one in the bilateral cerebellum and one in the right inferior parietal lobule.

For the Language by Priming interaction contrast [L1 (Unprimed – Primed) – L2 (Unprimed – Primed)], two significant clusters were found bilaterally in the posterior insula. For the reverse contrast, no significant clusters were found. To informally inspect whether the lack of significant activations was due to thresholding issues, the threshold was lowered to *p* < 0.005 (uncorrected). With this threshold, clusters were found in the pars opercularis of the LIFG and the left insula. However, they were too small (*k* < 7) to satisfy the corrected *p* < 0.05 threshold.

When L1 speakers were analyzed separately, the Unprimed vs. Primed contrast revealed no significant clusters. Again, to rule out thresholding issues, the threshold was lowered to *p* < 0.005 (uncorrected). The only cluster coming close to significance at this threshold was located in the left posterior superior temporal sulcus (*k* = 39). For the reverse contrast, no significant clusters were found either. At the *p* < 0.005 (uncorrected) threshold, small clusters were found in the right superior temporal gyrus, the left cerebellum, the right inferior parietal lobule, the right inferior frontal sulcus and left periventricular white matter. However, they were all too small to satisfy the corrected *p* < 0.05 threshold (*k* < 14).

In contrast, L2 speakers showed significant activation for the Unprimed vs. Primed contrast (see **Figure 2**). A large left-lateralized cluster stretched from the pars orbitalis over the pars triangularis to the pars opercularis of the LIFG (overlapping with the three LIFG ROIs), reaching into the insula. With the threshold lowered to *p* < 0.005 (uncorrected), the only other cluster coming close to significance was situated in the left supramarginal gyrus (*k* = 39). For the reverse contrast, significant bilateral clusters were found in the superior temporal gyrus, extending into the ventral insula, and in the dorsal insula, reaching into the right parietal operculum. Another significant right-lateralized cluster stretched from the parahippocampal gyrus into the hippocampus.

To summarize, the whole-brain analysis confirmed a clear Priming effect in the LIFG over both groups and for L2 participants, and revealed additional clusters of activation in bilateral temporal, parietal, and frontal regions over both groups.

## DISCUSSION

In this long-lag priming fMRI study, the processing of semantically transparent derived verbs was investigated in L1 and L2 speakers. The priming paradigm allowed us to determine whether the LIFG showed a repetition suppression effect to primed compared to unprimed transparent derivations. Such an effect would indicate that, in the LIFG, the primed target (derivation) is processed more efficiently because the same process has already been applied to the prime (stem). Since long-lag priming is supposed to reflect morphological rather than semantic or formal processing, this facilitation should be due to morphological decomposition rather than to semantic and/or form similarities between stem and derivation (see Introduction). Both ROI analyses and whole-brain analyses revealed that repetition suppression effects were indeed present in the LIFG for primed compared to unprimed complex verb targets. This was true both for the analyses over the two language groups together and for the analyses of L2 participants only. When L1 speakers were analyzed separately, no such priming effect was found. However, no evidence was found of a difference between the two language groups in the LIFG, as shown by the lack of a Language by Priming interaction in this area. The whole-brain analysis over both groups also revealed additional repetition suppression effects in mainly left-lateralized temporal, parietal, and frontal regions, and increased activations or repetition enhancement effects for primed compared to unprimed derived verbs in bilateral temporal and cerebellar regions and right parietal areas.

The involvement of the LIFG in morphological processing has been revealed in many neuroimaging studies on derivational and inflectional processing in L1 and L2 speakers, both in studies using a priming paradigm (L1 derivations: Bozic et al., 2007; Bick et al., 2009; L2 derivations: Bick et al., 2010; L2 inflections: Pliatsikas et al., 2014a) and in studies not using a priming paradigm (L1 derivations: Vannest et al., 2005, 2011; Meinzer et al., 2009; Pliatsikas et al., 2014b; L1 inflections: Laine et al., 1999; Tyler et al., 2005; Lehtonen et al., 2006; L2 inflections: Lehtonen et al., 2009; – for a discussion of the potential effect of using a priming paradigm, see below). The involvement of the LIFG has been interpreted as evidence for decomposition of morphologically complex words. More specifically, the LIFG has been postulated to be involved in morpho-phonological segmentation of complex words (Tyler et al., 2005). In another account (Lehtonen et al., 2006), however, this segmentation function is attributed to more posterior areas, such as the left occipitotemporal cortex (OT), whereas the LIFG is supposed to support later combinatorial processes in which stem and affix are phonologically and semantically integrated. This account is supported by studies suggesting that the LIFG is involved in controlled retrieval and manipulation processes of semantic and phonological representations (e.g., Poldrack et al., 1999; Wagner et al., 2001). In addition, several masked priming fMRI studies on morphological processing showed repetition suppression in the left OT for morphologically related word pairs, suggesting that this region is involved in early stages of morphological processing (L1 derivations: Gold and Rastle, 2007; L2 derivations: Bick et al., 2010; L2 inflections: Lehtonen et al., 2009). In our study, we did not find any involvement of the OT. This may be related to our use of long-lag priming, which may not be as sensitive to early effects as masked priming.

Left inferior frontal gyrus involvement in morphological processing is sometimes accompanied by the involvement of the left or bilateral posterior superior temporal gyrus (pSTG; L1 derivations: Meinzer et al., 2009; Vannest et al., 2011; Bozic et al., 2013; L1 inflections: Laine et al., 1999; Tyler et al., 2005) or superior temporal sulcus (pSTS; L1 inflections: Lehtonen et al., 2006). So far, this has only been found in studies on L1 morphological processing. The pSTG has been associated with phonological and/or lexico-semantic processing (phonological: Binder et al., 2009; Graves et al., 2014; lexico-semantic: Grindrod et al., 2008; Ruff et al., 2008; Ulrich et al., 2013), whereas activation of the pSTS has mainly been found for phonological processing (Price, 2000; Buchsbaum et al., 2001; Turkeltaub et al., 2003). The involvement of these areas in morphological processing has been attributed to lexical access to the stems of inflected words (Tyler et al., 2005) or access to semantic, phonological and/or syntactic representations of stems and affixes (Lehtonen et al., 2006).

In the current study, the priming paradigm led to a pattern of repetition suppression and repetition enhancement effects in both inferior frontal and posterior temporal areas for primed compared to unprimed morphologically complex words: repetition suppression effects were found in the LIFG and left pSTS, and repetition enhancement effects were found in bilateral pSTG. According to Henson (2003), repetition suppression indicates that the same type of processing occurs for primed and unprimed stimuli in the areas showing this effect, a processing that is facilitated by the prime in the primed condition, but not in the unprimed condition (for a more elaborate explanation, see Introduction). In contrast, repetition enhancement effects are generally interpreted to show additional processing for primed compared to unprimed stimuli in the areas showing increased activation (Henson, 2003). First, we will discuss the repetition suppression effects we found; then we will go into the repetition enhancement effects.

The repetition suppression effect in the left pSTS indicates that the (phonological) representations of the stems are accessed for both primed and unprimed transparent verbs, but that this is facilitated for the former because their stems have already been accessed upon presentation of the stem primes. The controlled retrieval account of the LIFG (e.g., Poldrack et al., 1999) suggests that the LIFG controls access to these representations, following decomposition of the complex verb into stem and affix (Lehtonen et al., 2006). In this account, the repetition suppression effect found in the LIFG indicates that controlled retrieval of the representations of stem and affix occurs for both primed and unprimed complex verbs, but that this is facilitated for the former because the stem representation is already retrieved upon presentation of the prime. The alternative account, i.e., that the LIFG supports the morphological segmentation process itself (Tyler et al., 2005), seems more difficult to integrate with the repetition suppression results. The facilitation reflected by repetition suppression is supposed to be due to performance of the same process on the prime as on the primed stimulus (Henson, 2003). Therefore, presentation of the stem prime should not lead to facilitation of the morphological segmentation process of the primed complex verb, as morphological segmentation is not performed on the stem prime itself. Of course, the LIFG may support morphological segmentation for both primed and unprimed complex verbs to a similar degree, in addition to controlling access to stem representations. This cannot be determined on the basis of the current study, as our results are dependent on the comparison of primed and unprimed complex verbs.

Next, we turn to the repetition enhancement effect. The increased activation in the bilateral pSTG indicates that additional semantic and/or phonological processing occurs for primed compared to unprimed complex verbs (see above). One could hypothesize, first, that priming of the stem can also lead to increased competition between the representation of the stem and the representation of the complex verb, and/or additional comparison processes between these representations. It is unclear, though, why this would not also lead to repetition enhancement effects in (subregions of) the LIFG, as the latter is supposed to control such processing.

Alternatively, the repetition enhancement effect in the pSTG may be related to learning. Repetition enhancement rather than repetition suppression effects have been found to occur with unfamiliar stimuli (Segaert et al., 2013). The repetition of unfamiliar stimuli may lead to the creation of new representations, which involves increased activation. In contrast, familiar stimuli already have stable representations, so that no increased activation is necessary to build their representations. The stimuli used in the current experiment were moderately frequent (approximately 13 per million). Thus, they would be familiar enough for L1 speakers, but probably relatively unfamiliar for L2 speakers, as also reflected by their relatively high error percentage. As shown in the whole-brain analyses, the repetition enhancement effects in our analysis over both groups seem to be primarily driven by the L2 speakers’ results. In fact, the only significant interaction between Language and Priming is due to repetition enhancement effects in the bilateral posterior insula in L2 speakers and not L1 speakers. Activation in this area has been related to (bilingual) language learning (Ardila et al., 2014). The presence of repetition enhancement effects in the right hippocampal and parahippocampal regions also seems to support the learning account, as activation in these areas may indicate that memory encoding is taking place (e.g., Stark and Okado, 2003).

The studies on morphological processing discussed so far have all found evidence for decomposition of transparent derivations by revealing the involvement of the LIFG (sometimes combined with the pSTS/STG) in their processing. In contrast, in some (non-priming) fMRI studies on L1 speakers, either no evidence for decomposition or evidence for holistic processing of transparent derived words was found. Davis et al. (2004) found no significant differences between transparent derived or inflected words vs. simple words. Bozic et al. (2013) reported increased activation in bilateral frontotemporal regions for opaque derivations (e.g., *archer*, *breadth*) and transparent unproductive derivations (e.g., *warmth*) compared to simple words (but not for transparent productive derivations (e.g., *bravely*) compared to simple words). This bilateral activation pattern (including LIFG and RIFG) was interpreted to reflect more general perceptual and semantic processes supporting language comprehension. Since no specific left-lateralized system was engaged, (transparent and opaque) derived words were supposed to be processed holistically. In contrast, inflected words were argued to be decomposed (Bozic et al., 2010), because they were processed by such a left-lateralized frontotemporal system (including LIFG but not RIFG), supposedly specialized for grammatical computations. In the present study, a repetition suppression effect was found in the LIFG and no effects were found in the RIFG for derived verbs. According to the account proposed by Bozic et al. (2010, 2013) this would be an indication that the transparent derived verbs were decomposed.

Several explanations can be provided for the discrepancy between our results (involvement of LIFG but not RIFG) and Bozic et al.’s (2013) results (involvement of both LIFG and RIFG). Firstly, we used a morphological priming paradigm, whereas Bozic et al. (2013) used direct comparisons between simple and complex words. Possibly, priming increases the probability that derived words are decomposed: presentation of the stem may increase the chance that the morphological structure of subsequently presented derived words is recognized. This explanation is supported by the results of Bozic et al. (2007). In the latter fMRI study, a long-lag priming paradigm was used with derived words, and a left-lateralized effect was found: a repetition suppression effect in the LIFG and not in the RIFG. The idea that priming may lead to increased decomposition is in line with results showing that the processing of derived words is influenced by factors affecting the recognition of their morphological structure. For example, derived words with longer suffixes tend to be decomposed rather than being processed holistically (Kuperman et al., 2010).

Another factor which may influence the processing of derived words is the choice of task. Like Bozic et al. (2007), Meinzer et al. (2009), and Pliatsikas et al. (2014b), we used a linguistic task (lexical decision), whereas Bozic et al. (2013) used a non-linguistic task (detection of silent gaps within auditory stimuli). Possibly, the lexical decision task directs attention more to the morphological structure of derived words than gap detection does.

So far, we have only discussed the analyses over both groups. These revealed a pattern of repetition suppression and repetition enhancement effects in LIFG and pSTS/STG. In contrast, the analyses over L1 speakers only did not show any significant effects. It is difficult to draw any conclusions from this, however, as no significant Language by Priming interactions were found in the left frontotemporal regions which are normally associated with morphological processing (LIFG and left posterior temporal cortex). Thus, no evidence was found of a difference between L1 and L2 speakers in terms of derivation processing. Also, the Bayesian analysis of the L1 ROI data only revealed weak evidence in favor of the null hypothesis of no priming in L1 speakers. Finally, in the pSTS, a cluster was found just below significance for L1 speakers, which did reach significance in the analysis over both groups. As mentioned above, the pSTS has also been associated with morphological processing. One possible explanation for the absence of a clear priming effect in L1 speakers may be related to the familiarity of our stimuli. As mentioned before, unfamiliar stimuli often elicit repetition enhancement effects, whereas familiar stimuli generally elicit repetition suppression effects. For the L1 speakers, our stimuli were moderately familiar, i.e., they may have been too familiar to elicit repetition enhancement effects, but not familiar enough to elicit clear repetition suppression effects. However, stimulus familiarity cannot account for the whole pattern of results, as L2 participants, for whom the stimuli were relatively unfamiliar, displayed both repetition enhancement and repetition suppression effects.

In contrast with L1 speakers, L2 participants did display clear priming effects in the LIFG. This suggests that L2 speakers do decompose transparent derived verbs, rather than relying on holistic processing. This confirms some of the previous results on morphological processing in L2 speakers (Bick et al., 2010; Diependaele et al., 2011; Pliatsikas et al., 2014a), but contrasts with other studies (Silva and Clahsen, 2008; Clahsen and Neubauer, 2010). As mentioned in the Introduction, however, none of these studies used an unmasked priming paradigm, which may explain the differences found with this study. (For a further discussion of whole-brain analysis results, see Supplementary Material, Further Discussion of Whole-Brain Results).

Besides the significant repetition suppression effect in the LIFG, L2 speakers also displayed a significant behavioral effect: more errors were made to unprimed than to primed complex verbs. However, the regression analysis we conducted showed that there is no indication that the neural priming effect found for L2 speakers is due to the increased difficulty of unprimed compared to primed complex verbs.

A limitation of the present study is that, due to the high degree of relatedness between Dutch and German, we could not use non-cognate verbs as stimuli (see Materials section). Therefore, our conclusions only pertain to the processing of cognate derivations by L2 speakers. Cognates have a special status in bilingual language processing, as they are not only similar in meaning in two languages, but also similar in form. The so-called “cognate facilitation effect” (e.g., Dijkstra et al., 2010) has shown that there might be transfer from L1 to L2 through cognates, at least in simple word recognition. It is not clear whether this special status also holds for morphological processing, and it remains to be investigated whether the same results are obtained for non-cognate as for cognate derived verbs. For this, a different language pair should be used, for example French L2 speakers of Dutch, so that enough non-cognate stimuli can be selected. Also, since our stimuli contained more particle (separable) verbs than prefixed (non-separable) verbs, the results we obtained may primarily have been driven by the particle verbs. However, as mentioned before, studies comparing the processing of particle and prefixed verbs have found no differences between the two types (Schriefers et al., 1991; Lüttmann et al., 2011). Therefore, we have no reason to assume that results would have been different if only prefixed verbs had been included.

To conclude, the central result of the present study is that L2 speakers of Dutch (with German as their L1) show a repetition suppression effect in the LIFG when processing semantically transparent derived Dutch verbs primed by their stems. In the context of other studies on the processing of morphologically complex words in L1 speakers, this indicates that German L2 speakers of Dutch decompose such morphologically complex verbs. In the whole-brain analysis over both L1 and L2 speakers of Dutch, the involvement of the LIFG was supplemented by a repetition suppression effect in the pSTS. This suggests that the (phonological) representations of the stems of the derivations are accessed after morphological decomposition, with the LIFG possibly controlling access to these stem representations. Additionally, L2 speakers of Dutch showed repetition enhancement effects in the bilateral superior temporal gyrus and insula and in the right parahippocampal gyrus. These may be related to L2 language learning, as the presentation of relatively unfamiliar stimuli may lead to the creation of new representations. Future research should address the question whether, first, the sensitivity of L2 speakers to morphological structure is restricted to morphologically complex words of the type investigated in this study, i.e., prefix verbs, or also generalizes to other types of morphologically complex words, such as suffixed nouns; and second, whether this morphological sensitivity of L2 speakers is restricted to languages with a similarly rich morphological system, such as Dutch and German (Basnight-Brown et al., 2007; Portin et al., 2008), or also generalizes to other language pairs (Pliatsikas et al., 2014a).

## Conflict of Interest Statement

The authors declare that the research was conducted in the absence of any commercial or financial relationships that could be construed as a potential conflict of interest.

## SUPPLEMENTARY MATERIAL

The Supplementary Material for this article can be found online at: http://www.frontiersin.org/journal/10.3389/fnhum.2014.00802/abstract

## Footnotes

1 We only review L1 behavioral studies in which a similar method and similar stimuli are used as in the present study, i.e., unmasked visual priming and transparent derivations. In contrast, our review of the L1 fMRI and L2 literature also includes studies in which other methods and/or stimuli are used, as there are hardly any fMRI and/or L2 studies on the processing of transparent derivations using unmasked visual priming.

2 Parts 1 and 2 of the fMRI session took place immediately after each other. In between the two parts, participants could take a small break of several minutes, during which they remained in the scanner.

3 In part 1 (De Grauwe et al., 2014), 22 L1 participants took part. One of them only participated in part 1 and not in part 2, resulting in 21 initial L1 participants for the current study.
