# Supplementary material for: L2 speakers decompose morphologically complex verbs: fMRI evidence from priming of transparent derived verbs
Source: Front Hum Neurosci. 2014 Oct 10;8:802. doi: 10.3389/fnhum.2014.00802 (PMC4193264; doi:10.3389/fnhum.2014.00802)
Supplement: Supplementary file 1 [file Data_Sheet_1.DOCX]

***Supplementary Material***

**L2 speakers decompose morphologically complex verbs: fMRI evidence from priming of transparent derived verbs**

**Sophie De Grauwe^1*^, Kristin Lemhöfer^1^, Roel M. Willems^1,2^, Herbert Schriefers^1^**

^1^Radboud University Nijmegen, Donders Institute for Brain, Cognition and Behavior, Nijmegen, Netherlands

^2^Max Planck Institute for Psycholinguistics, Nijmegen, Netherlands

*** Correspondence:** Sophie De Grauwe, Radboud University Nijmegen, Postbus 9104, 6500 HE Nijmegen, Netherlands.

s.degrauwe@donders.ru.nl

1. **Supplementary Tables**

**Table S1. Mean ratings.** Self-assessment of Dutch proficiency by L2 speakers.

| **Frequency reading Dutch** | **Frequency speaking Dutch** | **Frequency listening to/ watching Dutch radio/TV** | **Total reading experience Dutch** | **Total writing experience Dutch** | **Total speaking experience Dutch** |
| --- | --- | --- | --- | --- | --- |
| 5.24 (1.48) | 5.52 (1.25) | 2.95 (1.94) | 5.19 (1.12) | 4.90 (1.04) | 5.19 (1.17) |

*Notes. Rating scale: 1-7. Standard deviations in parentheses.*

**Table S2. Stimulus characteristics.**

|  | **Complex Verbs** | | **Stems** | |
| --- | --- | --- | --- | --- |
|  | *Primed* | *Unprimed* | *Primes* | *Fillers* |
| **Length** | 8.94 (1.26) | 9.14 (1.40) | 6.31 (1.11) | 6.43 (1.38) |
| **Frequency** | 1.00 (0.41) | 0.96 (0.47) | 2.15 (0.59) | 2.29 (0.74) |
| **Transparency** | 3.61 (0.60) | 3.51 (0.59) | - | - |
| **Motor-Relatedness** | - | - | 2.69 (1.22) | 2.56 (1.17) |

*Notes. Length: number of letters.*

*Frequency: log-transformed raw frequency numbers.*

*Transparency: rated on a scale of 1-5 (1 = low degree, 5 = high degree of transparency).*

*Motor-Relatedness: rated on a scale of 1-5 (1 = low degree, 5 = high degree of motor-relatedness).*

*Means are shown with standard deviations in parentheses.*

*-: Not applicable.*

**Table S3. Experimental stimuli.**

| *Prime*  *(Stem)* | *Primed Target (Complex Verb)* | *Separable?* |
| --- | --- | --- |
| breken  dragen  drukken  hangen  leggen  schrijven  vangen  geven  steken  strijken  graven  wijzen  klemmen  schroeven  wikkelen  wegen  sluiten  nemen  dekken  horen  rekenen  rusten  scheiden  zorgen  leven  helpen  tellen  merken  laten  schijnen  wonen  vallen  passen  dienen  zweren | afbreken  meedragen  aandrukken  ophangen  wegleggen  opschrijven  opvangen  teruggeven  insteken  bestrijken  opgraven  aanwijzen  vastklemmen  vastschroeven  omwikkelen  afwegen  afsluiten  meenemen  bedekken  aanhoren  uitrekenen  uitrusten  afscheiden  verzorgen  samenleven  meehelpen  aftellen  bemerken*  loslaten  beschijnen  bewonen  omvallen  aanpassen  bedienen  afzweren^+^ | yes  yes  yes  yes  yes  yes  yes  yes  yes  no  yes  yes  yes  yes  yes  yes  yes  yes  no  yes  yes  yes  yes  no  yes  yes  yes  no  yes  no  no  yes  yes  no  yes |

*Notes.* *: excluded in the L1 data analysis;

^+^: excluded in the L2 data analysis.

| *Unprimed Target (Complex Verb)* | *Separable?* |
| --- | --- |
| opvreten  uitroepen  toespreken  nazeggen  meegaan  meekomen  weglopen  opspringen  opstaan  betreden  omkeren  besnuffelen  wegbrengen  aanduiden  afwenden  vasthouden  afmeten  terugdringen  omvatten  afsterven  inoefenen*^+^  uitbloeien^+^  afwisselen  inslapen  beantwoorden  uitzoeken  betwijfelen  opschrikken  aanvoelen  doordenken  terugvinden  weerzien  bestralen  misgunnen*  overbieden | yes  yes  yes  yes  yes  yes  yes  yes  yes  no  yes  no  yes  yes  yes  yes  yes  yes  no  yes  yes  yes  yes  yes  no  yes  no  yes  yes  yes  yes  yes  no  no  no |

**Table S4. Whole-brain analyses.** List of significant activations.

|  | **Both groups** |  |  | **L1** |  |  | **L2** |  |  |
| --- | --- | --- | --- | --- | --- | --- | --- | --- | --- |
| *Contrast* | *Brain Region* | *MNI x y z* | *Nr Vox* | *Brain Region* | *MNI x y z* | *Nr Vox* | *Brain Region* | *MNI x y z* | *Nr Vox* |
| Unprimed – | LIFG: pars orbitalis | -42 30 -6 | 347 |  |  |  | LIFG: pars orbitalis | -40 30 -10 | 1454 |
| Primed | LIFG: pars opercularis | -60 16 18 | 259 |  |  |  | LIFG: pars opercularis | -58 14 20 |  |
|  | L supramarginal gyrus | -54 -42 26 | 153 |  |  |  | *L supramarginal gyrus* | *-52 -36 36* | *39* |
|  | L superior temporal sulcus | -56 -32 4 | 105 | *L superior temporal sulcus* | *-58 -32 6* | *39* |  |  |  |
|  | L & R medial superior frontal gyrus | -12 8 70  6 12 58 | 161 |  |  |  |  |  |  |
| Primed –  Unprimed | L insula/superior temporal gyrus | -46 -14 0 | 145 |  |  |  | L superior temporal gyrus  L insula | -48 -14 0  -42 0 -8 | 477 |
|  | R superior temporal gyrus | 38 -50 14 | 70 |  |  |  | R superior temporal gyrus/insula | 48 -8 -2 | 919 |
|  | R parahippocampal gyrus/hippocampus | 34 -20 -18 | 96 |  |  |  | R parahippocampal gyrus | 32 -22 -20 | 173 |
|  | L & R cerebellum | -4 -40 -14  4 -40 -12 | 65 |  |  |  | L dorsal insula | -34 -26 20 | 89 |
|  | R inferior parietal lobule | 42 -46 40 | 92 |  |  |  | R dorsal insula | 34 0 16 | 124 |
| Interaction: L1 (uP – P) –  L2 (uP – P) | L insula | -38 -20 2 | 75 | - |  |  | - |  |  |
|  | R insula | 38 -12 4 | 82 | - |  |  | - |  |  |
|  |  |  |  |  |  |  |  |  |  |

*Notes*. Listing of activations for the main effect of Priming and the Language by Priming interaction in the whole-brain analyses. MNI-coordinates of the maximally activated voxels are shown. Correction for multiple comparisons at p < .05 resulting from the combination of a voxel-level p-value of p <.005 (uncorrected) with a minimum cluster size of 65 voxels ([Forman et al., 1995](#_ENREF_2); [Slotnick, Moo, Segal, and Hart Jr, 2003](#_ENREF_5)). Italics used for clusters just below this threshold. Nr Vox: number of voxels; L: left-hemisphere; R: right-hemisphere; uP: Unprimed; P: Primed; LIFG: left inferior frontal gyrus; -: not applicable.

1. **Supplementary Information**

## Further discussion of whole-brain results

The whole-brain analysis over both groups revealed additional effects in frontal, parietal and cerebellar regions. Repetition suppression effects were found in the left supramarginal gyrus (SMG) and the bilateral pre-supplementary motor area (pre-SMA). The left SMG has been associated with phonological processing ([Jobard, Crivello, and Tzourio-Mazoyer, 2003](#_ENREF_3); [Xu et al., 2001](#_ENREF_8)), although some evidence of its involvement in semantic processing has been found, too ([Stoeckel, Gough, Watkins, and Devlin, 2009](#_ENREF_6)). The whole-brain analyses of the two language groups separately suggest that the repetition suppression effect in the left SMG may have been primarily driven by the L2 speakers, as they showed a cluster in this region just below significance. As a reminder, the L1 speakers showed a repetition suppression effect that was just below significance in the left pSTS. Possibly, there is a shift in the importance of the left pSTS to the left SMG for phonological processing by L2 speakers compared to L1 speakers. This is speculative, though, since there was no interaction between Priming and Language in these areas.

The pre-SMA has been found to be involved in higher-level planning of motor activity (e.g., Picard and Strick, 1996). The repetition suppression effect in this area suggests that responses to primed compared to unprimed complex verbs were facilitated due to the previous presentation of the stem prime. Thus, this effect seems to be the result of response priming rather than morphological priming.

Further repetition enhancement effects were found in the bilateral cerebellum and the right inferior parietal lobule (IPL). The cerebellum has been shown to be involved in language processing, such as semantic, phonological and morphological processing ([De Smet, Paquier, Verhoeven, and Mariën, 2013](#_ENREF_1)). Support for the involvement of the cerebellum in morphological processing comes from several fMRI studies on inflections (Laine, Rinne, Krause, Teräs, and Sipilä, 1999; Pliatsikas, Johnstone, and Marinis, 2014).

Finally, the right IPL has been associated with semantic processing, more specifically the creation of meaning from distant associations ([Subramaniam, Faust, Beeman, and Mashal, 2012](#_ENREF_7)). The repetition enhancement effect in this area could perhaps be due to more elaborated semantic integration for primed compared to unprimed transparent derivations.

1. **References**

De Smet, H. J., Paquier, P., Verhoeven, J., and Mariën, P. (2013). The cerebellum: Its role in language and related cognitive and affective functions. *Brain Lang.* 127, 334-342. doi: 10.1016/j.bandl.2012.11.001

Forman, S. D., Cohen, J. D., Fitzgerald, M., Eddy, W. F., Mintun, M. A., and Noll, D. C. (1995). Improved assessment of significant activation in functional magnetic resonance imaging (fMRI): Use of a cluster-size threshold. *Magn. Reson. Med.* 33, 636-647. doi: 10.1002/mrm.1910330508

Jobard, G., Crivello, F., and Tzourio-Mazoyer, N. (2003). Evaluation of the dual route theory of reading: A metanalysis of 35 neuroimaging studies. *NeuroImage* 20, 693-712. doi: 10.1016/S1053-8119(03)00343-4

Laine, M., Rinne, J. O., Krause, B. J., Teräs, M., and Sipilä, H. (1999). Left hemisphere activation during processing of morphologically complex word forms in adults. *Neurosci. Lett.* 271, 85-88. doi: 10.1016/S0304-3940(99)00527-3

Picard, N., and Strick, P. L. (1996). Motor areas of the medial wall: A review of their location and functional activation. *Cereb. Cortex* 6, 342-353. doi: 10.1093/cercor/6.3.342

Pliatsikas, C., Johnstone, T., and Marinis, T. (2014). fMRI evidence for the involvement of the procedural memory system in morphological processing of a second language. *PLoS ONE* 9: e97298. doi: 10.1371/journal.pone.0097298

Slotnick, S. D., Moo, L. R., Segal, J. B., and Hart Jr, J. (2003). Distinct prefrontal cortex activity associated with item memory and source memory for visual shapes. *Cogn. Brain Res.* 17, 75-82. doi: 10.1016/s0926-6410(03)00082-x

Stoeckel, C., Gough, P. M., Watkins, K. E., and Devlin, J. T. (2009). Supramarginal gyrus involvement in visual word recognition. *Cortex* 45, 1091-1096. doi: 10.1016/j.cortex.2008.12.004

Subramaniam, K., Faust, M., Beeman, M., and Mashal, N. (2012). The Repetition Paradigm: Enhancement of novel metaphors and suppression of conventional metaphors in the left inferior parietal lobe. *Neuropsychologia* 50, 2705-2719. doi: 10.1016/j.neuropsychologia.2012.07.020

Xu, B., Grafman, J., Gaillard, W. D., Ishii, K., Vega-Bermudez, F., Pietrini, P., . . . Theodore, W. (2001). Conjoint and extended neural networks for the computation of speech codes: The neural basis of selective impairment in reading words and pseudowords. *Cereb. Cortex* 11, 267-277. doi: 10.1093/cercor/11.3.267
